# Hypotension, Syncope, and Fever in Systemic Mastocytosis without Skin Infiltration and Rapid Response to Corticosteroid and Cyclosporin: A Case Report

**DOI:** 10.1155/2010/782595

**Published:** 2010-12-20

**Authors:** Didem Ozdemir, Selcuk Dagdelen, Tomris Erbas, Kemal Agbaht, Songul Serefhanoglu, Salih Aksu, Sibel Ersoy-Evans

**Affiliations:** ^1^Department of Internal Medicine, Hacettepe University Medical School, Sıhhiye, 06350 Ankara, Turkey; ^2^Department of Endocrinology and Metabolism, Hacettepe University Medical School, 06350 Ankara, Turkey; ^3^Department of Hematology, Hacettepe University Medical School, 06350 Ankara, Turkey; ^4^Department of Dermatology, Hacettepe University Medical School, 06350 Ankara, Turkey

## Abstract

Mast cell disorders are defined by an abnormal accumulation of tissue mast cells in one or more organ systems. In systemic mastocytosis, at least one extracutaneous organ is involved by definition. Although, systemic mastocytosis usually represents with skin lesion called urticaria pigmentosa, in a small proportion, there is extracutaneous involvement without skin infiltration. Other manifestations are flushing, tachycardia, dyspepsia, diarrhea, hypotension, syncope, and rarely fever. Various medications have been used but there is not a definite cure for systemic mastocytosis. The principles of treatment include control of symptoms with measures aimed to decrease mast cell activation. We describe a case of systemic mastocytosis presenting with hypotension, syncope attacks, fever, and local flushing. In bone marrow biopsy, increased mast cell infiltration was demonstrated. She had no skin infiltration. A good clinicopathological response was obtained acutely with combination therapy of glucocorticoid and cyclosporine.

## 1. Introduction

Mastocytosis is a rare hematopoietic disorder which is characterized by abnormal proliferation and accumulation of mast cells in one or more organs [1]. Mast cell disorders are recently included under the category of myeloproliferative neoplasms by the 2008 World Health Organization classification of myeloid neoplasms [2]. Mastocytosis limited to the skin is called cutaneous mastocytosis, and when extracutaneous organs such as bone marrow, liver, spleen, or gastrointestinal tract are involved, it is called systemic mastocytosis (SM) [3]. Cutaneous mastocytosis is largely a disease of infancy and childhood, while SM is usually seen in adults. Systemic mastocytosis which accounts for about 10% of all cases of mastocytosis is a persistent disease that can follow benign or indolent course or may be associated with hematological disorders. Major clinical manifestations of SM are episodic flushing, dyspepsia, diarrhea, abdominal pain, tachycardia, and pruritus [4]. These are related directly to tissue infiltration or to the release of mast cell mediators like leukotrienes and histamine. 

Here, we report an SM patient presenting with flushing, hypotension, fever, and syncope attacks. Her symptoms have been successfully controlled and mast cell infiltration in bone marrow decreased significantly after short-term corticosteroid and cyclosporine treatment.

## 2. Case Report

**Figure 1:**
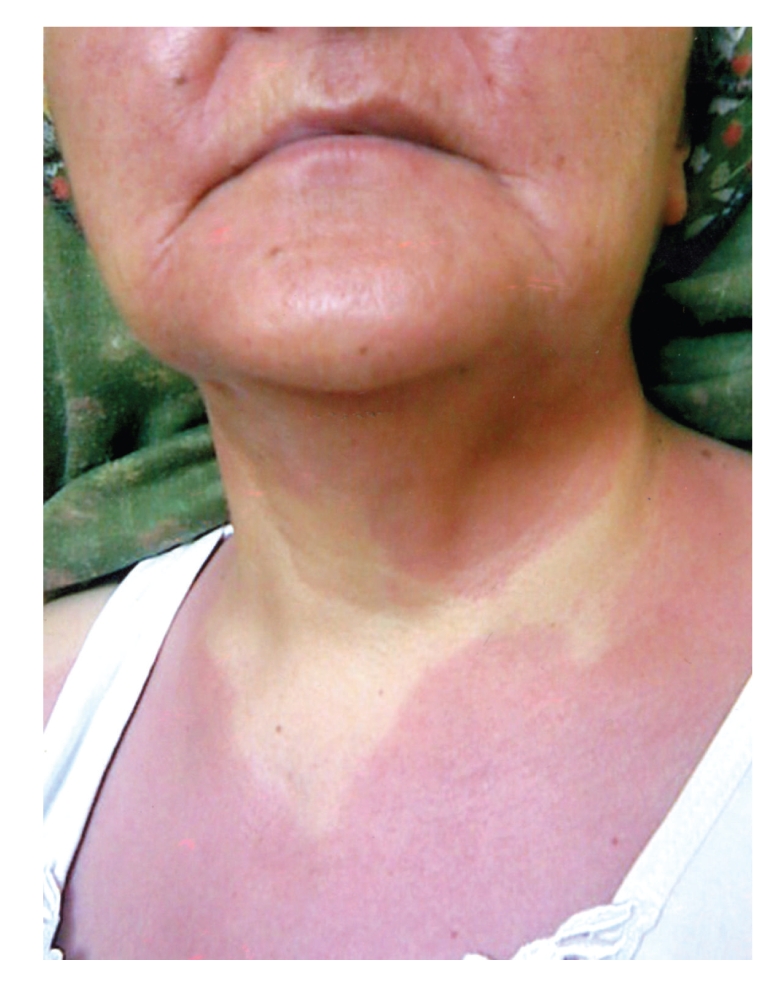
Flushing in face, neck, and upper chest with sharp margins.

**Figure 2:**
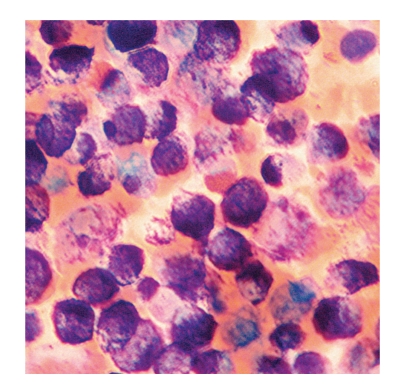
Infiltration with mast cells in bone marrow aspiration (original magnification ×1000).

A 52-year-old woman presented with fatigue, flushing, dyspepsia, hypotension, and syncope attacks for about 18 months. She defined headache and fatigue at the beginning of attack, and then flushing occurred in her face and neck primarily, descending to body but not to extremities. During attacks, she had hypotension with systolic blood pressure between 50–70 mmHg, diastolic blood pressure between 20–40 mmHg, and followed by 5–10 minutes of syncope. While flushing resolved in about an hour, she sometimes had diarrhea, dyspepsia, nausea, and vomiting before or just after the attacks. After she had experienced fever reaching 39°C in the last attack, she was referred to our hospital. In the 11th day of hospitalization, she developed nausea, vomiting, and fever of 40°C ongoing with severe hypotension (systolic and diastolic blood pressures of 50 mmHg and 25 mmHg, resp.). She had conjunctival hyperemia and flushing in her face, neck, and upper chest with sharp margins (Figure 1). She was transferred to critical care unit for close monitorization and management of hemodynamic instability. With intravenous fluid replacement therapy, her blood pressure increased up to 120/70 mmHg and hemodynamic stability was achieved in an hour. There was no pneumonic infiltration and blood and urine cultures were negative for any microorganism. Thoracoabdominal computed tomography was normal. Transesophageal echocardiography revealed no vegetation. Carcinoid syndrome and pheochromocytoma were excluded based on normal urine catecholamines and 5-hydroxyindoleacetic acid checked before and during the first 4 hours of the attack. Bone marrow aspiration and biopsy were performed because of anemia (hemoglobin = 6.9 mg/dl) and thrombocytopenia (110.000/*μ*L) (Table 1). Bone marrow aspiration showed that 80% of nucleated cells were mast cells (Figure 2), and biopsy revealed a hypercellular bone marrow infiltrated with mast cells. In addition, c-kit staining was positive in bone marrow biopsy, immunohistochemically. Serum tryptase and histamine levels at the time of attack were >200 ng/mL (<13.5 ng/mL) and >100 nmol/L (<10 nmol/L), respectively. Her serum hemoglobin and platelet concentrations returned to acceptable levels without any transfusion in a few days (hemoglobin = 8.9 g/dl and platelets = 176.000/*μ*L).

She was administered on prednisolone (1 mg/kg/day) and cyclosporine (300 mg/d) with a H1 receptor blocking agent (desloratadine 5 mg/day). After esophagogastroduodenoscopy, an H2 receptor blocking agent (famotidine 80 mg/day) was also initiated since she had duodenal ulcer. After three weeks of corticosteroid and cyclosporine treatment, repeated bone marrow biopsy showed normal morphology and in aspiration mast cells constituted 15% of nucleated cells. Serum tryptase level was still >200 ng/mL; however, histamine level decreased to 29.7 nmol/L. About 10 weeks after she was discharged from the hospital, Fip1-like1 (FIP1L1) gene mutation analysis was found to be negative and she was administered on interferon alpha treatment. She was free of any attacks or other symptoms for about 5 months and in the last visit her serum hemoglobin and platelet levels were 10.9 g/dl and 238.000/*μ*L, respectively.

## 3. Discussion

Diagnosis of SM is confirmed with evidence of involvement of a tissue other than skin, most commonly bone marrow, spleen, liver, lymph node, and gastrointestinal tract. Main symptoms related to mast cell degradation are episodic flushing caused by vasodilatation, dyspepsia, nausea, vomiting, diarrhea, and abdominal pain. There may be associated hypotension and syncope due to cerebral hypoperfusion. Such an acute attack typically lasts from 15 to 30 minutes and may be precipitated by a variety of triggers such as physical exertion, emotional upset, heat, cold, ethanol, intravenous contrast exposure, and certain medications (nonsteroidal anti-inflammatory drugs, opioids, and general anesthetics). In our patient, attacks of flushing, hypotension, and syncope with nonspecific symptoms like fatigue, diarrhea, nausea, vomiting, and dyspepsia were all suggestive for SM. Although fever is not a known classical symptom for SM, there are limited numbers of cases that presented with fever in the literature [5, 6]. Our patient also had a body temperature reaching 39°C in her last two severe attacks. 

Skin lesions may or may not accompany SM. Although urticaria pigmentosa which is known to be the characteristic lesion of mastocytosis was described in 80% of SM patients previously [7], recently Lim et al. showed that 41% of patients with SM had urticaria pigmentosa and 53% had cutaneous symptoms including pruritus, flushing, urticaria, or angioedema [8]. It is considered that more aggressive forms of SM which have unfavorable prognosis are more likely to present without cutaneous lesions [9]. There was no skin lesion except intermittent and temporary flushing and urticarial rash in our patient, and Darier' sign was negative. Diffuse erythema during attacks were supposed to result from histamine release rather than skin infiltration by mast cells. 

The major criterion for diagnosis of SM is the finding of multifocal dense infiltrates of mast cells in bone marrow or other extracutaneous tissues. There are also 4 minor criteria defined as follows: (a) atypical mast cell morphology, (b) aberrant mast cell surface immunophenotype, (c) serum total tryptase >20 ng/mL, and (d) c-kit mutation at codon 816 in extracutaneous organs [10]. If at least one major and one minor criterion or at least three minor criteria are fulfilled, the final diagnosis of SM can be confirmed. Tryptase which is stored almost exclusively within the secretory granules of mast cells is the most widely used marker of mastocytosis. In healthy individuals, serum tryptase levels range between <1 and 15 ng/mL; however, mast cell activation causes increased tryptase levels [11]. Additionally, tryptase levels in SM are assumed to correlate closely with the cumulative mast cell burden and multiorgan involvement [5]. Mast cell infiltration of bone marrow has been shown in our patient. Additionally, high serum tryptase (>200 ng/mL) levels and positive c-kit staining in bone marrow biopsy confirmed our diagnosis. However, we could not make analysis for D816V c-kit mutation due to technical reasons.

World Health Organization classify 7 types of mastocytosis: cutaneous mastocytosis, indolent SM, SM with an associated clonal hematological nonmast cell lineage disease, aggressive SM, mast cell leukemia, mast cell sarcoma, and extracutaneous mastocytoma [12]. Cutaneous mastocytosis is an indolent disease which can only be diagnosed when SM is excluded by appropriate investigations. The most common variant of SM is indolent SM which is differentiated from more advanced categories of SM by lack of end organ dysfunctions and relatively low infiltration grade. Aggressive SM is characterized by evidence of end organ dysfunction such as significant cytopenia, ascites, malabsorption, splenomegaly, or pathologic fractures due to osteolysis. If mast cells comprise >20% of all nucleated cells in bone marrow aspirate and are increased in circulation with ≥10% mast cells in peripheral blood, this is called mast cell leukemia. SM in our patient was subtyped as mast cell leukemia as 80% of bone marrow aspirate was composed of mast cells. 

Modalities used in the treatment of SM are directed to two targets: symptomatic control and decrease in mast cell burden. Commonly used medications for symptomatic relief are H1 and H2 antihistamines, oral disodium cromoglycate, and epinephrine for hypotensive episodes. However, currently there is no cure for more serious types of SM. Interferon alpha is the drug for which most experience has been reported, but there are conflicting results about it [13–15]. Combination of interferon alpha with corticosteroid has also been shown to have a beneficial effect in controlling symptoms of SM [16]. Cladribine and imatinib mesylate are other two cytoreductive agents recently used for SM patients and showed promising results [17, 18]. It was found that patients with a gene translocation resulting in fusion of the FIP1L1 gene and platelet-derived growth factor (PDGF) receptor alpha genes respond well to imatinib mesylate [19]. However, the major problem with that drug is that patients that are positive for D816V c-kit mutation are usually resistant to its effects [20]. In the literature, we found one patient with aggressive SM showing a good response to cyclosporine and corticosteroid treatment [21]. We also treated our patient with corticosteroid and cyclosporine in the early period. Bone marrow biopsy after three weeks of this therapy showed significant reduction in mast cell burden. Besides, the patient was nearly asymptomatic after a few days of therapy. In followup, based on negative FIP1L1-PDGFR mutation analysis, she was administered on interferon alpha treatment.

## 4. Conclusion

Systemic mastocytosis without skin involvement may represent with attacks of flushing, hypotension, syncope, and fever mimicking septic shock or cardiovascular collapse. Combination therapy with corticosteroid and cyclosporin seems to help to control symptoms and decrease mast cell burden in a short time.

##  Conflict of Interests

Authors declare that there is no conflict of interests that could be perceived as prejudicing the impartiality of this paper.

## Figures and Tables

**Table 1:** Laboratory data at first admission, during attack and after treatment.

	At the time of hospitalization	During attack	3 weeks after treatment
Complete blood count:			
Hemoglobin (12–18 gr/dl)	9.5	6.9	11.9
Hematocrit (36–54%)	27.5	18.8	33.5
Erythrocyte (3.5–6.0 × 10^6^/mcl)	2.97	2.02	3.24
White blood cells (3600–10000/mcl)	13600	9100	11200
Neutrophile (37–75%)	86.3	72.0	73.7
Lymphocyte (20–55%)	8.1	9.0	22.0
Monocyte (2.5–10%)	1.0	4.7	2.2
Eosinophil (0.5–11%)	4.6	5.7	2.1
Basophil (0–2%)	0.0	8.6	0.0
Platelet (150.000–450.000/mcl)	286.000	110.000	234.000
Erythrocyte sedimentation rate (0–20 mm)	33	7	21
Tryptase (<13.5 ng/mL)	—	>200	>200
Histamine (<10 nmol/L)	—	>100	29.7
